# Ambrine

**Published:** 1917-04

**Authors:** 


**Ambrine** (sold as Parresine in this country). E. Callamand reports observing several severe cases with the same astonishing results described by Dr. Wm. O'Neill Sherman of Pittsburg, before the Buffalo Academy of Medicine (Le Moniteur Med., Feb. 13). H. D'Arcy Power, Calif. State Jour., March, refers to Barthe's report of the ambrine treatment of burns and of frost bites, etc., and himself applied the treatment to a case of ulceration following diabetic gangrene. He used $3\frac{1}{2}$ parts each of white wax and paraffin and 1 part of resin, filtering through cheese cloth and applying with a brush. Ambrine is also applied in a heated spray. All accounts emphasize the complete freedom from pain.

